# Case report: Supratherapeutic tacrolimus concentrations with nirmatrelvir/ritonavir in a lung transplant patient: a case report using Rifampin for reversal

**DOI:** 10.3389/fphar.2023.1285078

**Published:** 2023-11-10

**Authors:** Yu Xiong, Xiaoxing Wang, Shu Li, Qian Zhang, Lijuan Guo, Wenhui Chen, Zhixia Zhao, Lihong Liu

**Affiliations:** ^1^ Institute of Materia Medica, Chinese Academy of Medical Sciences and Peking Union Medical College, Beijing, China; ^2^ Department of Pharmacy, China-Japan Friendship Hospital, Beijing, China; ^3^ Department of Pulmonary and Critical Care Medicine, China-Japan Friendship Hospital, Beijing, China; ^4^ Clinical Trial Research Center, China-Japan Friendship Hospital, Beijing, China

**Keywords:** PAXLOVID, nirmatrelvir/ritonavir, tacrolimus toxicity, drug-drug interactions, Rifampin

## Abstract

Paxlovid (nirmatrelvir/ritonavir) is an antiviral drug used to treat COVID-19, nirmatrelvir, a SARS-CoV-2 main protease inhibitor, works by inhibiting viral replication in the early stages, and ritonavir is a strong cytochrome P450 (CYP) 3A inhibitor that helps the nirmatrelvir reach and maintain the therapeutic concentrations. Paxlovid has a potential risk of drug interaction by elevating the plasma concentration of other drugs metabolized by CYP3A, like tacrolimus. This report examines the case of a 57-year-old female lung transplant patient self-administered Paxlovid for 5 days without discontinuing tacrolimus. She presented to the hospital with symptoms of headache, dizziness, palpitations, abdominal distension, nausea, vomiting, and diarrhea. The patient presented with tacrolimus toxicity and the blood concentration of tacrolimus was measured at 106 ng/mL. Urgent medical intervention was initiated, and Rifampin was administered to induce enzyme activity and rapidly decrease the concentration of tacrolimus. By adjusting the tacrolimus dosage, the final concentration was brought within the appropriate range. Clinical pharmacists should prioritize medication education for transplant patients to prevent severe drug interactions and minimize the impact on the patient’s overall well-being.

## 1 Introduction

In the past 3 years, the coronavirus 2019 disease (COVID-19) continued to disrupt global health and challenged the healthcare systems globally, and China also experienced an increase in morbidity and mortality (World Health Organization, 2022). The launch of Paxlovid (Nirmatrelvir Tablets/Ritonavir Tablets (co-packaged)) has significantly reduced the rates of hospitalization and mortality (Hammond et al., 2022; Wong et al., 2022). Nirmatrelvir is a peptidomimetic inhibitor of the SARS-Cov-2 major protease Mpro (also known as 3C-like protease, 3CLpro), and inhibition of SARS-CoV-2 Mpro prevents it from processing the multiprotein precursor, thereby preventing viral replication (Owen et al., 2021). Nirmatrelvir is easily and rapidly metabolized by the hepatic enzyme CYP3A4 and is therefore co-administered with the irreversible CYP3A4 inhibitor ritonavir. Ritonavir, an old anti-HIV drug, is used as a pharmacokinetic enhancer to help the nirmatrelvir reach and maintain the therapeutic concentrations. Due to the use of ritonavir, nirmatrelvir/ritonavir has potential threats to cause many drug-drug interactions (DDIs) with other concurrent medications, especially for drugs metabolized by CYP3A, such as Clopidogrel, Ticagrelor, Tacrolimus, Simvastatin, etc (Hammond et al., 2022).

Lung transplant patients, who require lifelong administration of tacrolimus, have become a prioritized population for monitoring and managing the use of Paxlovid during the COVID-19 pandemic. Tacrolimus, a calcineurin inhibitor (CNI), is widely utilized in the treatment of solid organ transplant patients, including kidney, lung, and liver transplants, as well as individuals with autoimmune and hematological disorders. However, its narrow therapeutic window, proximity between therapeutic and toxic doses, and substantial interindividual variability pose significant challenges in its administration (Scalea et al., 2016; Schutte-Nutgen et al., 2018). The therapeutic efficacy of tacrolimus is highly dependent on achieving and maintaining optimal blood concentrations. Consequently, careful attention is required when utilizing tacrolimus in clinical practice (Yu et al., 2018).

Tacrolimus is primarily metabolized by the hepatic and intestinal cytochrome P450 (CYP) enzyme systems, with CYP3A4 serving as the key enzyme, followed by CYP3A5. Additionally, tacrolimus is a substrate of the efflux transporter P-glycoprotein (P-gp), which influences its absorption in the small intestine. Consequently, drugs that compete with tacrolimus for CYP3A metabolism, affect CYP3A4/5 enzyme activity, or modulate P-gp-mediated transport can potentially alter the pharmacokinetic profile of tacrolimus, leading to changes in its blood concentrations (Yu et al., 2018). Elevated blood concentrations lead to a range of adverse reactions, including renal toxicity, neurotoxicity, electrolyte derangements, and electrocardiographic abnormalities such as prolonged QT interval (Farouk and Rein, 2020).

Many cases have reported that the concomitant use of Paxlovid and tacrolimus can lead to acute kidney injury (AKI) and other adverse reactions in the transplant population (Shah et al., 2022; Sindelar et al., 2023; Tsuzawa et al., 2023). In this report, we present a case of a lung transplant patient lacking appropriate professional guidance, self-medicated with Paxlovid following SARS-CoV-2 infection without adjusting the dose of tacrolimus. The patient presented with tacrolimus toxicity (106 ng/mL), rifampicin was subsequently used as a CYP3A4 inducer to quickly reduce the tacrolimus concentration. The purpose of this case report is to investigate the management of DDIs between Paxlovid and tacrolimus in lung transplant patients. And we hope to provide more clinical warnings and more clinical evidence for the combination of tacrolimus and Paxlovid.

## 2 Case description

We present a case report of a 57-year-old female who underwent bilateral lung transplantation in 2017 for connective tissue disease-associated interstitial lung disease and Sjögren’s syndrome. Her medical history included hypertension following long-term corticosteroid use, chronic gastritis, gastroesophageal reflux disease, vertebral compression fracture, severe osteoporosis, lacunar brain infarction, abnormal liver function, and hyperlipidemia. The patient’s outpatient tacrolimus regimen was 2 mg every morning and 2 mg every evening, with a target tacrolimus level of 4–6 ng/mL. The patient self-administered Paxlovid for 5 days after testing positive for SARS-CoV-2 antigen and nucleic acid, following a week of intermittent fever (37.4°C). On the sixth day, she presented to the emergency department (ED) with symptoms of headache, dizziness, palpitations, abdominal distension, nausea, vomiting, and diarrhea when she finished Paxlovid.

**FIGURE 1:**
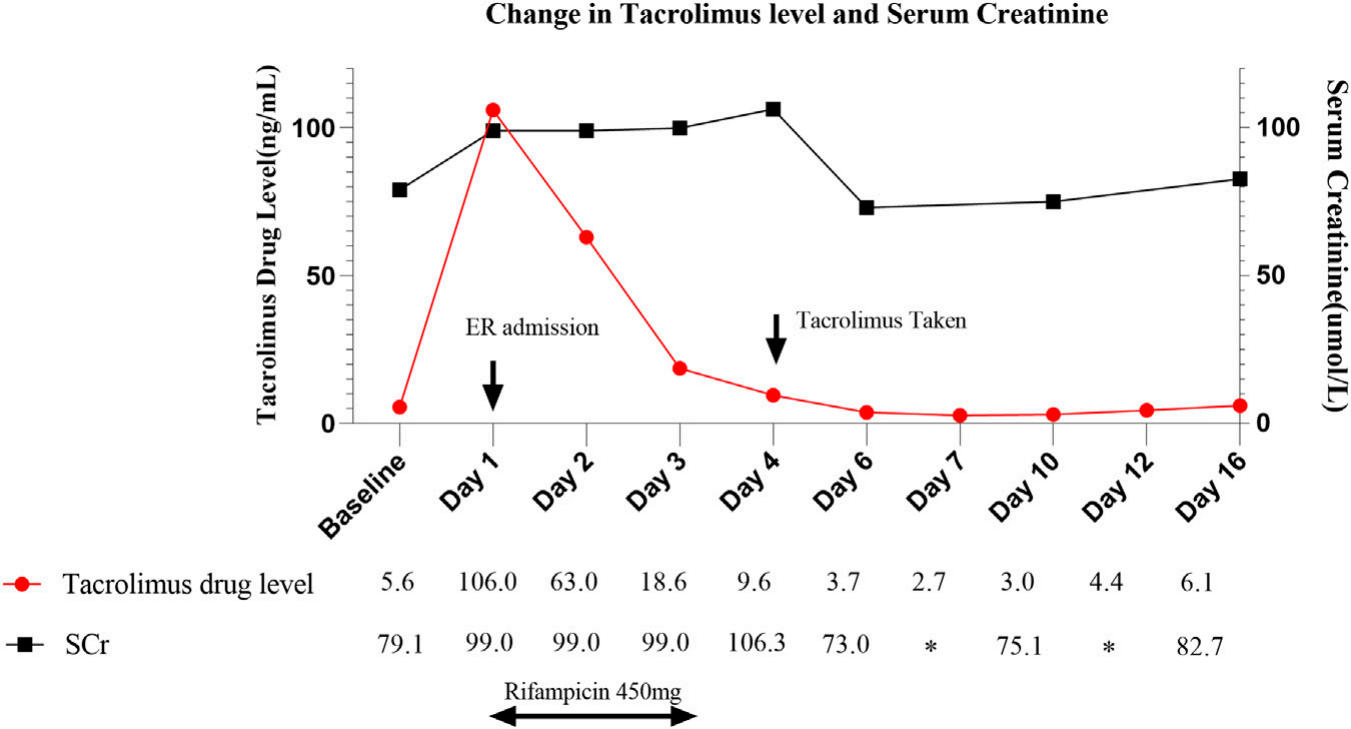
Change in tacrolimus level in correlation to trend in serum creatinine and rifampicin administration throughout hospitalization. ER, Emergency Room.

Upon admission, the patient’s tacrolimus level was found to be 106 ng/mL, accompanied by evident signs of tacrolimus toxicity, including drowsiness, vivid dreams, and prolonged QT interval, despite normal creatinine levels. Tacrolimus was discontinued and our clinical team initiated oral rifampicin at a dose of 450 mg once daily, utilizing its CYP3A4-inducing properties. Our team observed a nearly 50% decrease in tacrolimus concentration on the first day of rifampicin administration and an approximately 25% decrease on the second day. Considering the delayed onset of enzyme induction, it was speculated that the tacrolimus concentration may return to the normal range on the fourth day. Therefore, on the morning following discontinuation of rifampicin, the patient was administered 1.5 mg of tacrolimus once daily. However, therapeutic drug monitoring (TDM) revealed persistently elevated tacrolimus levels (9.6 ng/mL), prompting further dose adjustments. Eventually, it took 6 days for her tacrolimus level to decrease to 3.7 ng/mL, after that, the patient’s tacrolimus concentration stabilized within the appropriate therapeutic range (6.1 ng/mL). Fortunately, the patient’s renal function was not much affected, and the creatinine value was always within the normal range, unlike other cases where AKI was caused by high tacrolimus concentrations (Stawiarski et al., 2023), showing large individual differences in tacrolimus (Steinman, 2016). Tacrolimus concentrations and creatinine values are shown in Figure 1.

## 3 Discussion

DDIs occur when the activity of one drug is altered due to the presence of another drug, taken simultaneously or consecutively. DDIs often lead to unexpected pharmacological effects, exposing patients to greater risks of adverse effects, toxicity, and even life-threatening situations (Steinman, 2016). According to previous research reported, unintentional adverse drug events (ADEs) cause over 770,000 cases of harm (hospitalizations, ICU visits, etc.) and deaths annually, with approximately 50% of ADEs attributed to DDIs (Ibrahim et al., 2016). In this case, the DDIs between tacrolimus and Paxlovid resulted in tacrolimus toxicity, while the DDIs between rifampicin and tacrolimus caused decreased concentration.

When faced with the case, the main goal was to expediently decrease the tacrolimus blood concentration and alleviate the associated toxicity. Furthermore, precise monitoring of therapeutic drug levels is required for the safe readministration of tacrolimus to prevent potential rejection reactions. Tacrolimus toxicity should be addressed primarily through supportive therapy since hemodialysis is ineffective due to tacrolimus’s large volume of distribution and being 99% protein bound (Sindelar et al., 2023). A more practical approach is to accelerate tacrolimus metabolism using enzyme induction. The pharmacist reviewed prior literature on combining tacrolimus and Paxlovid and assessed clinical experience with the medication (e.g., our team had prior clinical experience with a rifampicin dosage of 300 mg to reduce slightly elevated levels of tacrolimus in the blood). Eventually, rifampicin 450 mg once daily was selected as the enzyme-inducing agent, and it is worth noting that this dosage is safe for treating *mycobacterium tuberculosis* infections in solid organ transplantation (Subramanian et al., 2019). Before commencing rifampicin treatment, we undertook a DDIs check with MicromedexⓇ to ensure that the patient’s other medications were being used appropriately. The patient underwent a course of rifampicin administered at a daily dose of 450 mg for 3 days, following which the concentration of tacrolimus was monitored with long-term therapeutic drug monitoring (TDM) (Gervasoni et al., 2015). By the sixth day, the tacrolimus concentration had returned to normal levels. This observation is consistent with other case studies (Rose et al., 2022), but their common dosages of rifampicin used are 300 mg or 600 mg once daily, and 600 mg twice daily was also reported in some cases (Rose et al., 2022).

Why does it generally take around 5–7 days for tacrolimus toxicity reactions to decrease to normal levels? This is because enzyme induction refers to the process where a substance (inducer) increases the synthesis or activation of enzymes, thereby enhancing their activity. Inducers typically modulate gene expression or alter the conformation of enzymes to increase their quantity or modify their activity. Since this involves biological processes such as gene expression and protein synthesis, it requires time to occur. Conversely, enzyme inhibition occurs rapidly. Enzyme inhibition occurs when a substance (inhibitor) binds to an enzyme, thereby reducing its activity. Inhibitors can bind to the active site of the enzyme, preventing substrate binding or catalytic reactions, or they can bind to other sites and alter the enzyme’s conformation, rendering it inactive. The process of enzyme inhibition is generally swift, as the binding between the inhibitor and the enzyme typically involves a rapid physical or chemical interaction (Barry and Feely, 1990). Therefore, the concentration of tacrolimus increases rapidly, but its decrease is slow.

Both rifampicin and phenytoin can be used as enzyme inducers to lower tacrolimus concentration. Phenytoin is commonly used overseas to lower tacrolimus blood concentrations. The typical duration of phenytoin administration is 3–4 days, taking about 6 days to reach the safe therapeutic range, the same as rifampicin (Kwon et al., 2022; Shah et al., 2022; Sindelar et al., 2023). Phenytoin is primarily metabolized by CYP enzymes, which may make it particularly susceptible to inhibitory drug interactions, resulting in increased bioavailability. On the other hand, the pharmacokinetics of phenytoin is difficult to predict, and adverse reactions may occur. Another factor regarding adverse reactions is the relatively long half-life of phenytoin, which averages around 42 h after oral administration (Hoppe et al., 2022). In contrast, rifampicin is an effective CYP inducer with a half-life of approximately 2–3 h. Therefore, its potent CYP-inducing properties, short half-life, and CYP-independent elimination support its use, especially in patients presenting severe signs of tacrolimus toxicity, including organ dysfunction. However, phenytoin may have an advantage in patients experiencing severe neurological symptoms (such as seizures and convulsions) caused by tacrolimus toxicity (Hoppe et al., 2022).

Regarding the issue of DDIs between Paxlovid and tacrolimus, it inevitably leads to an increase in tacrolimus concentration. Since tacrolimus is a long-term medication for lung transplant patients, even if tacrolimus is discontinued during Paxlovid administration, the inhibitory effect of the enzyme immediately takes effect, inevitably affecting the steady-state concentration of tacrolimus. Therefore, self-administration of Paxlovid and tacrolimus without clinical pharmacists monitoring poses significant risks, even varying degrees of impact on the lives of transplant patients. Thus, careful monitoring for DDIs should be conducted when prescribing Paxlovid for patients with COVID-19 pneumonia, especially in transplant patients with tacrolimus. This case highlights the significance of a “patient-centered” model of healthcare in clinical practice. Clinical pharmacists in the field of transplantation should effectively serve both the clinic and patients by providing comprehensive medication education. Conducting effective patient education, including ensure regular follow-up, and preparing medication instruction lists that clarifies necessary precautions, such as DDIs that need to be cautioned against, etc. Clinicians, clinical pharmacists, and patients must work together to prevent such incidents from recurring.

In addition, the self-purchase and administration of Paxlovid outside the hospital was a considerable factor in this medical event, indicating that the ethical implications of self-administration of medication deserve deeper consideration. Patients have the right to make informed decisions about their healthcare. However, it is essential to ensure that the patient is fully informed about the risks and benefits of self-administration and that they have the capacity to make this decision. Self-administration of medication without medical guidance can pose significant risks. Therefore, this requires that clinicians and clinical pharmacists make sure to provide complete and unbiased information about medications, potential consequences, and available alternatives. And, it is ethically imperative to stress the importance of seeking medical guidance and supervision. Patients should be encouraged to consult with healthcare professionals who can provide expertise and oversight.

Some limitations remain with this case report. Relying only on the experience of a single lung transplant patient, as well as the lack of long-term follow-up data, so larger sample sizes and more case studies are needed to establish generalizable findings.

## Data Availability

The original contributions presented in the study are included in the article/Supplementary material, further inquiries can be directed to the corresponding authors.
